# Synchrotron radiation-based quasi-elastic scattering using time-domain interferometry with multi-line gamma rays

**DOI:** 10.1038/s41598-017-12216-7

**Published:** 2017-10-02

**Authors:** Makina Saito, Ryo Masuda, Yoshitaka Yoda, Makoto Seto

**Affiliations:** 1 Research Reactor Institute, Kyoto University, Kumatori-cho, Sennan-gun, Osaka 590-0494 Japan; 2 Research and Utilization Division, Japan Synchrotron Radiation Research Institute, Sayo-cho, Sayo-gun, Hyogo 679-5198 Japan

## Abstract

We developed a multi-line time-domain interferometry (TDI) system using 14.4 keV Mössbauer gamma rays with natural energy widths of 4.66 neV from ^57^Fe nuclei excited using synchrotron radiation. Electron density fluctuations can be detected at unique lengths ranging from 0.1 nm to a few nm on time scales from several nanoseconds to the sub-microsecond order by quasi-elastic gamma-ray scattering (QGS) experiments using multi-line TDI. In this report, we generalize the established expression for a time spectrum measured using an identical single-line gamma-ray emitter pair to the case of a nonidentical pair of multi-line gamma-ray emitters by considering the finite energy width of the incident synchrotron radiation. The expression obtained illustrates the unique characteristics of multi-line TDI systems, where the finite incident energy width and use of a nonidentical emitter pair produces further information on faster sub-picosecond-scale dynamics in addition to the nanosecond dynamics; this was demonstrated experimentally. A normalized intermediate scattering function was extracted from the spectrum and its relaxation form was determined for a relaxation time of the order of 1 μs, even for relatively large momentum transfer of ~31 nm^−1^. The multi-line TDI method produces a microscopic relaxation picture more rapidly and accurately than conventional single-line TDI.

## Introduction

It is important to understand the microscopic dynamics in condensed matter when examining the physical properties and functionalities of systems. Quasi-elastic scattering measurements are performed extensively in microscopic dynamics studies because they provide the dynamics from a microscopic viewpoint. Shortly after the discovery of the Mössbauer effect, the Rayleigh scattering of Mössbauer radiation (RSMR) method, which is a quasi-elastic scattering method that uses a gamma ray probe from a radioactive isotope (RI), was first used^1^. In this method, 14.4 keV Mössbauer gamma rays that are emitted elastically from the first excited state of ^57^Fe nuclei with a natural energy width *Γ*_0_ of 4.66 neV are primarily used as probe photons. The momentum transfer ***q*** of the scattering process, which is determined by the scattered gamma rays detection angle, is related to the spatial scale ***l*** of the electron density in the form ***q*** ~ 2π/***l***. The energy broadening *Γ* (full width at half maximum: FWHM) in the energy spectrum due to quasi-elastic scattering by the sample reflects the electron density dynamics within the sample^1^. Dynamics with 100 ns time scales have been studied on both atomic and molecular length scales. However, RSMR measurements require very long periods (e.g. weeks), partly because the RI source emits gamma rays isotropically and only the proportion of the gamma rays that pass through a slit are used. A synchrotron radiation-based 14.4 keV Mössbauer radiation source (SMS) that uses a nuclear Bragg monochromator has been used as a source in quasi-elastic scattering experiments^2,3^. This SMS enables more efficient quasi-elastic scattering experiments because Mössbauer gamma rays emitted at the nuclear Bragg scattering angle inherit the directionality characteristics of synchrotron radiation (SR).

Recently, time-domain Mössbauer experiments have been widely performed using SR sources with pulsed time structures^4^. Similarly, SR-based time-domain quasi-elastic gamma-ray scattering (QGS) has been demonstrated via time-domain interferometry (TDI)^5–7^. We added the term “gamma-ray” to the original name of the QGS method to explicitly indicate the probe used for the quasi-elastic scattering^5^. In the TDI, two identical gamma ray emitters with a single excitation energy profile, e.g., stainless steel foil or K_2_MgFe(CN)_6_, are generally used to generate the probe and reference gamma rays. We consider the case where one of these gamma ray emitters is driven at a constant velocity relative to the other. When the gamma-ray energies from two emitters are separated appropriately by driving through the Doppler effect, their temporal interference pattern (called the quantum beat: QB) can be observed within the gamma-ray time spectrum^4,8^. The intermediate scattering function *S*(***q***, *t*) is obtained directly from the disappearance of the QB pattern, where *t* is time^5–7^. This is called the QB regime^7^. Conversely, if the separation of the gamma ray energies is relatively small when compared with their energy widths, the system response function cannot simply be written as per the QB regime case^7^. This regime is called the radiative coupling (RC) regime^7^.

Various dynamic studies have been performed using RSMR, QGS, and Mössbauer spectroscopy techniques to date (see supplementary note §I for examples). These studies clearly showed that the microscopic fluctuations in various condensed matters, including liquids, solids and soft matter, can all be observed on time scales of 100 ns by QGS. However, conventional QGS with 14.4 keV gamma rays from ^57^Fe nuclei uses a limited energy component of ~neV width in the white spectrum of SR (~eV) because gamma ray emitters with single-line excitation profiles are used. The gamma ray intensity is thus limited and conventional QGS requires relatively long measurement times, e.g. 10 h^9^. To overcome this problem, TDI systems were developed using double-line emitters, and the measurement efficiency was improved^10^. However, in both single-line and double-line TDI methods, unfavourable fluctuations in the constant Doppler velocity due to the mechanical motion of the velocity transducer lead to additional large-scale broadening of the energy widths of the probe gamma rays; this causes “pseudo-relaxation” in the resulting *S*(***q***, *t*). This broadening significantly reduces the potential efficiency of TDI, particularly when the sample’s dynamics are relatively slow (*Γ* < *Γ*_0_). Additionally, an adequate gamma ray count-rate, even in time regions longer than several 100 ns, is favourable for determination of the detailed *S*(***q***, *t*) form because the form of *S*(***q***, *t*) can be established more accurately by measuring over a wider time region. However, we found that increasing the number density of the nuclei in the emitters has only a limited impact on the measurement efficiency of the *S*(***q***, *t*) form because of the so-called speed-up effect, which suppresses the gamma ray count rate in the relatively delayed time region^10^.

The multi-line TDI method was developed to improve the measurement efficiency in QGS experiments^11^. In this method, hyperfine splitting of the nuclear energy levels in the gamma ray emitters, e.g. α-Fe foils, is used to create the nuclear excitation energy difference between the emitters rather than driving of the emitter. Therefore, the pseudo-relaxation effect is greatly suppressed. In addition, in the case of the multi-line method, increasing the number of gamma-ray lines via hyperfine interactions allows the speed-up effect to be suppressed in comparison to that which occurs in the case when single-line emitters with the same number density for the nucleus are used. Suppression of the speed-up effect is another reason why higher measurement efficiency has been demonstrated by the multi-line method to date when compared with that of the single-line method^11^.

For the purposes of extraction of *S*(***q***, *t*) from the spectrum and the determination of the *S*(***q***, *t*) form, we consider the QB regime, in which the time spectrum can be treated much more simply than the RC regime^7^. In this report, we generalize the expression for the time spectrum in the QB regime to the case of a nonidentical gamma ray emitter pair using incident SR with a finite energy width (typically ~meV). The equation obtained illustrates the unique characteristics of the quasi-elastic scattering time spectrum obtained by multi-line TDI. By studying glycerol, we obtained information about the dynamics on sub-ps and ns–μs scales, and the values obtained were consistent with previously obtained values. The normalized *S*(***q***, *t*) was also extracted from the spectrum and relaxation times of the order of 1 μs were obtained, even at a relatively high ***q*** ~ 31 nm^−1^.

## Expression for QGS time spectrum using TDI

**Figure 1:**
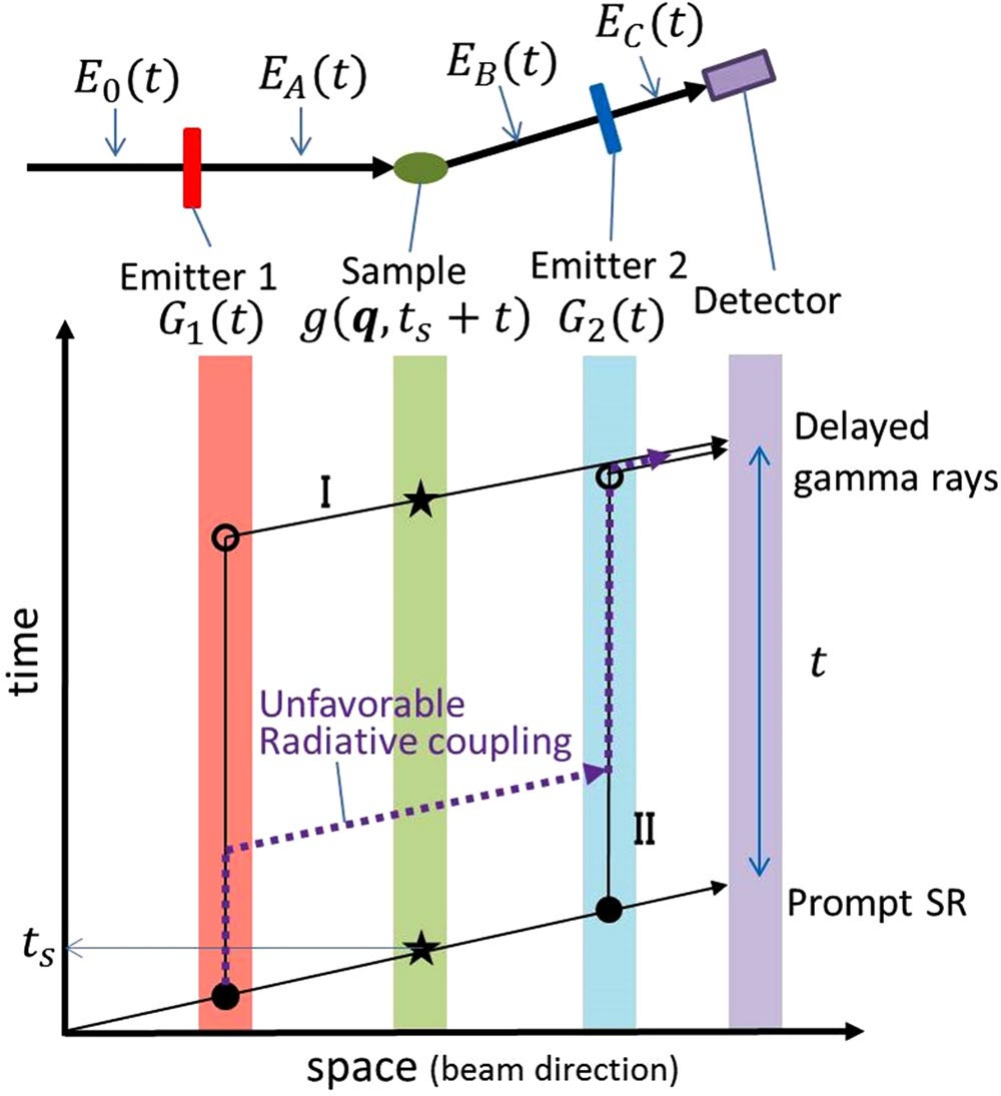
General views of QGS setups using TDI and time-space diagram of the gamma ray paths. Schematic general view of QGS setup using TDI and time-space diagram of the SR and gamma ray paths indicated by solid arrows in the setup. The paths of the gamma rays, which arrive at the detector with time delay *t* after the prompt SR, are shown as examples. Nuclear excitation occurs at the filled circle points, while de-excitation occurs at the empty circle points. The Thomson scattering process of the sample occurs at the star points. The dashed arrow indicates an example of the gamma ray path when unfavourable radiation coupling occurs.

**Figure 2:**
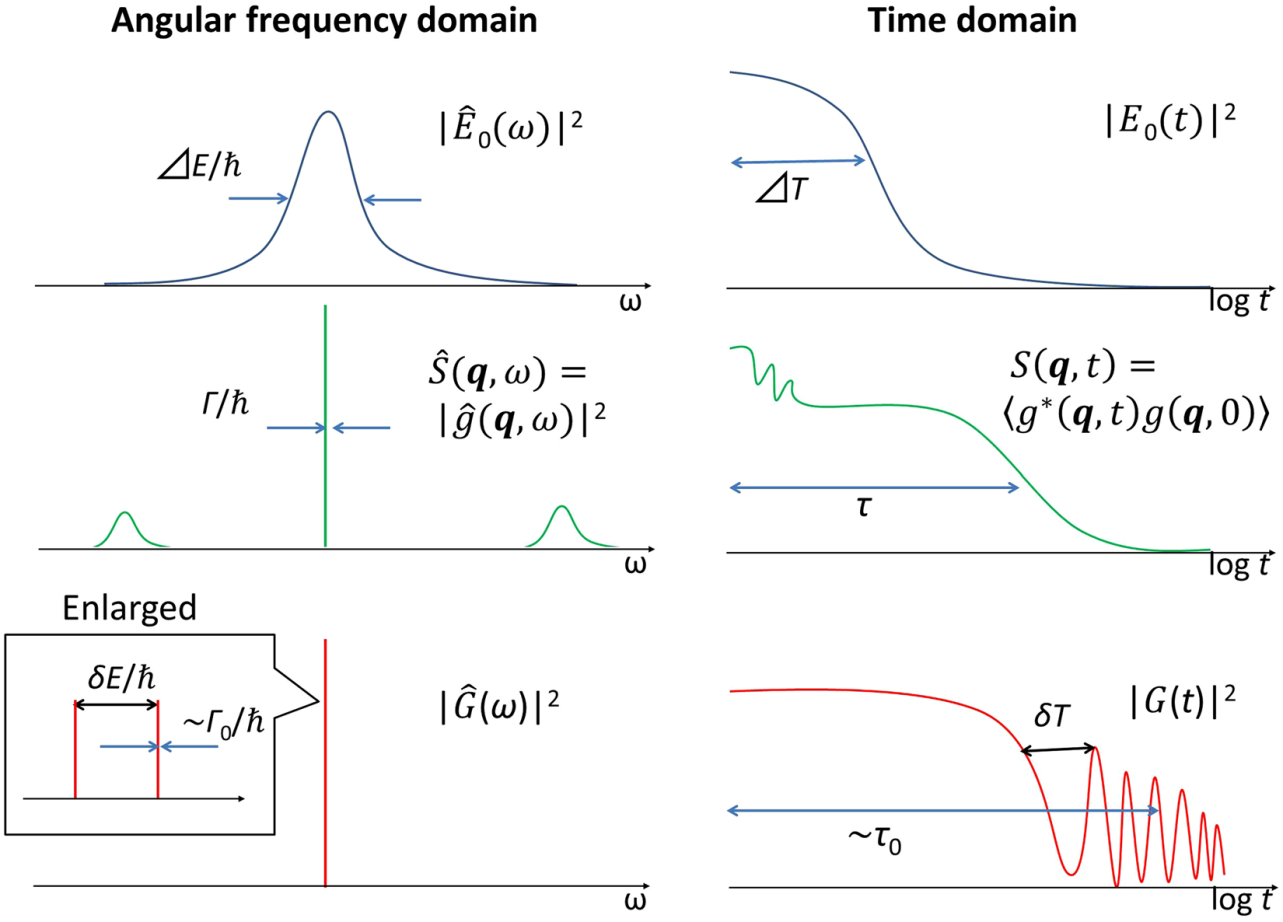
Examples of the incident SR spectrum, sample response function, and response function of the nuclei in the emitters. (Top) The spectrum in the angular frequency domain ${|{\hat{E}}_{0}(\omega )|}^{2}$ and the time spectrum |*E*_0_(*t*)|^2^ of the incident SR are shown. (Middle) The dynamic structure factor $\hat{S}({\boldsymbol{q}},\omega )={|\hat{g}({\boldsymbol{q}},\omega )|}^{2}$ is drawn, showing a central peak with FWHM of *Γ* and side-band peaks that originated from the lattice vibrations, where *g* is the response function of the sample (see the main text for details). The corresponding intermediate scattering function *S*(***q***, *t*) = 〈*g* ^*^(***q***, *t*)*g*(***q***, 0)〉 is also shown. Here, $\langle \cdots \rangle$ denotes a correlation function. (Bottom) For the response function of the nuclei in the emitters, the spectrum of ${|\hat{G}(\omega )|}^{2}$ in the angular frequency domain is shown for the condition with two peaks with energy splitting of *δE*. In the time domain, the corresponding |*G*(*t*)|^2^ showing the QB with a period of *δT* is drawn.

We consider the multi-line TDI setup shown in Fig. 1. The gamma-ray emitters (emitter 1 and emitter 2) are placed upstream and downstream of the (nonresonant) sample, respectively. The time-space diagram in Fig. 1 shows the paths of the incident SR and the gamma rays from the two emitters that were detected with time delay *t* after the prompt SR. We write the electric field amplitude of the incident SR in the angular-frequency (*ω*) domain as ${\hat{E}}_{0}(\omega )$ and the FWHM of ${|{\hat{E}}_{0}(\omega )|}^{2}$ as *ΔE*/*ħ*. The incident SR is generally monochromatized to the meV order around the nuclear excitation energy to reduce the quantity of unused radiation and prevent damage to both the sample and the detector. *E*_0_(*t*) is defined as the time-domain representation of ${\hat{E}}_{0}(\omega )$ and |*E*_0_(*t*)|^2^ decays on a time scale of *ΔT* ~ 2*ħ*/*ΔE* (see Fig. 2 for example spectra). A finite *ΔT* causes coherent broadening of the time width of the time spectrum. Note that incoherent time-spectrum broadening is also considered after the intensity of the time spectrum is obtained. We define *t* = 0 as the time when |*E*_0_(*t*)|^2^ reaches a maximum at the detector position. The response function of the emitter *i* is denoted by *R*_*i*_(*t*), and is expressed as ${R}_{i}(t)=\sqrt{{\rho }_{i}}\{\delta (t)-{G}_{i}(t)\}$ (*i*=1, 2), which includes the prompt term *δ*(*t*); here, *ρ*_*i*_ denotes the transmittance of the SR and gamma rays, while *G*_*i*_(*t*) denotes the time-delay component of *R*_*i*_(*t*) when defined as a nuclear response function^12–15^. We define the frequency-domain representation of *R*_*i*_(*t*) as ${\hat{R}}_{i}(\omega )$. The decay of |*G*_*i*_(*t*)|^2^ occurs over the time scale of a typical lifetime of excited nuclei *τ*_0_^4^. The FWHM of the peak(s) on the frequency-domain spectrum ${|{\hat{G}}_{i}(\omega )|}^{2}$, where ${\hat{G}}_{i}(\omega )$ was obtained through Fourier transformation of *G*_*i*_(*t*), is of the order of *Γ*_0_/*ħ* = 1/*τ*_0_^12,13^. Note here that the speed-up effect affects both the decay time of |*G*_*i*_(*t*)|^2^ and the width of ${|{\hat{G}}_{i}(\omega )|}^{2}$ ^16^. When several peaks are present on ${|{\hat{G}}_{i}(\omega )|}^{2}$ (*i* = 1, 2), the typical energy difference between peaks is denoted by *δE*. In the time-domain representation of |*G*_*i*_(*t*)|^2^, the typical QB time scale is *δT* = *h*/*δE*. We write the typical energy difference between the peaks of ${|{\hat{G}}_{1}(\omega )|}^{2}$ and those of ${|{\hat{G}}_{2}(\omega )|}^{2}$ as *δE*_12_. The typical time scale for the QB, which is affected by the dynamics, is denoted by *δT*_12_ = *h*/*δE*_12_. In the following discussion, we assume the QB regime, where $\Delta E\gg \delta {E}_{12}\gg {\Gamma }_{0}$ and $\delta {E}_{12}\gg \Gamma$, i.e., ${\tau }_{0}\gg \delta {T}_{12}\gg \Delta T$ and $\tau \gg \delta {T}_{12}$. We also assume that the incident SR pulse interval is sufficiently longer than *τ*_0_.

We obtain the equation for the time spectrum using the setup shown in Fig. 1. The electric field amplitude after emitter 1 is written as ${\hat{E}}_{A}(\omega )={\hat{R}}_{1}(\omega ){\hat{E}}_{0}(\omega )$ ^12,13^. Using the convolution theorem, the time-domain representation of this amplitude is ${E}_{A}(t)={R}_{1}(t)\otimes {E}_{0}(t)$, where $\otimes$ denotes a convolution. We define *t*_*s*_ = 0 as the time when |*E*_0_(*t*)|^2^ reaches a maximum at the sample position. The sample response is generally dependent on both *t*_*s*_ and *t*, while, conversely, the response of the nucleus is independent of *t*_*s*_^6,7^. The electric field amplitude after scattering by the sample is written as *E*_*B*_(*t*, *t*_*s*_) = *g*(***q***, *t*_*s*_ + *t*)*E*_*A*_(*t*), where the sample’s time response function is written as $g({\boldsymbol{q}},{t}_{s}+t)\propto \int d{\boldsymbol{r}}\,{{\rm{e}}}^{i{\boldsymbol{q}}\cdot {\boldsymbol{r}}}\rho ({\boldsymbol{r}},{t}_{s}+t)$ using the electron density *ρ*(***r***, *t*)^5^. Here, ***r*** denotes the position vector of the electrons. We write the frequency response function as $\hat{g}({\boldsymbol{q}},\omega )$, and $\hat{g}({\boldsymbol{q}},\omega )={\int }_{-\infty }^{\infty }dt\,{{\rm{e}}}^{i\omega t}g({\boldsymbol{q}},{t}_{s}+t)$. In the frequency domain, it then follows that ${\hat{E}}_{B}(\omega )=\hat{g}({\boldsymbol{q}},\omega )\otimes {\hat{E}}_{A}(\omega )$. After transmission through emitter 2, ${\hat{E}}_{c}(\omega )$ is written as ${\hat{E}}_{c}(\omega )={\hat{R}}_{2}(\omega ){\hat{E}}_{B}(\omega )$ in a similar manner to the case of emitter 1. It is then expressed in the time domain as *E*_*c*_(*t*, *t*_*s*_) = *R*_2_(*t*) ⊗ *E*_*B*_(*t*, *t*_*s*_). Finally, we obtain *E*_*c*_(*t*, *t*_*s*_) at the detector position in the form ${E}_{c}(t,{t}_{s})={R}_{2}(t)\otimes [g({\boldsymbol{q}},{t}_{s}+t)\,\{{R}_{1}(t)\otimes {E}_{0}(t)\}]$. This is an extension of the equation that was derived in refs ^5–7^, where we assumed that *R*_1_(*t*) = *R*_2_(*t*) and *E*_0_(*t*) = *δ*(*t*). The time-delay component when the RC is neglected is expressed as follows:

1 $$\begin{array}{rcl}{E}_{C}^{delay}({\boldsymbol{q}},t,{t}_{s}) & = & -\sqrt{{\rho }_{1}{\rho }_{2}}\,{\int }_{-\infty }^{\infty }dt^{\prime} g({\boldsymbol{q}},{t}_{s}+t)\,{G}_{1}(t-t^{\prime} )\,{E}_{0}(t^{\prime} )\\  &  & +{G}_{2}(t-t^{\prime} )\,g({\boldsymbol{q}},{t}_{s}+t^{\prime} )\,{E}_{0}(t^{\prime} ).\end{array}$$

The first and second terms in equation (1) are the amplitudes of the electric fields of the gamma rays that passed along paths I and II in Fig. 1, respectively. In the time-space diagrams of Fig. S1(a,b) in supplementary note §II, we show examples of the gamma rays from paths I and II when detected at *t* with incident times of *t*′ = 0 (long dashed lines) and *t*′ ≠ 0 (short dashed lines), respectively. In both gamma ray paths I and II, gamma rays with different incident times *t*′ interfere at the detector because of the finite coherent time width of |*E*_0_(*t*)|.

From the delayed electric field expressed in equation (1), the time spectrum $\bar{I}({\boldsymbol{q}},t)$ that is obtained by averaging based on *t*_*s*_ over the long measurement time *t*_*m*_ is expressed as equation (S5) in supplementary note §II. For convenience in the analysis shown below, we rewrite equation (S5) using the forward-scattering time spectra *I*_*i*_(*t*) ∝ |*G*_*i*_(*t*)|^2^ and ${I}_{1+{2}_{{\rm{coh}}}}(t)\propto {|{G}_{1}(t)+{G}_{2}(t)|}^{2}$ that were obtained when using emitter *i* and when using both emitters with the same measurement time, respectively. When we define *S*′(***q***, *t*) ≡ *S*(***q***, *t*)/*S*(***q***, 0), it then follows that

2 $$\begin{array}{ccc}\bar{I}({\boldsymbol{q}},t) & \propto  & \{1-{S}^{{\rm{^{\prime} }}}({\boldsymbol{q}},t)\}\,\{{\rho }_{2}{I}_{1}(t)+{\rho }_{1}{I}_{2}(t)\}+{S}^{{\rm{^{\prime} }}}({\boldsymbol{q}},t)\,{I}_{1+{2}_{{\rm{c}}{\rm{o}}{\rm{h}}}}(t)-\{1-{f}_{{\rm{\Delta }}E}({\boldsymbol{q}})\}\,{\rho }_{1}{I}_{2}(t)\end{array}$$

at *t* ≫ *ΔT*﻿﻿,﻿ where ${f}_{\Delta E}({\boldsymbol{q}})\equiv {\int }_{-\infty }^{\infty }dt^{\prime\prime\prime} {I}_{0}(t^{\prime\prime\prime})\,S({\boldsymbol{q}},t+t^{\prime\prime\prime} )/\,S({\boldsymbol{q}},0)$ using the time spectrum of the incident SR *I*_0_(*t*). See supplementary note §II for a further explanation of *f*_*ΔE*_(***q***). Because the measurements of *I*_1_(*t*) and *I*_2_(*t*) are performed using only one emitter, they must be multiplied by *ρ*_2_ and *ρ*_1_, respectively, to represent the existence of the other emitter in each case, as in the QGS and ${I}_{1+{2}_{{\rm{coh}}}}(t)$ measurement cases. Using equation (2), *S*′(***q***, *t*) can then be extracted by observing the shift from the coherent spectrum ${I}_{1+{2}_{{\rm{coh}}}}(t)$ towards the incoherent spectrum *ρ*_2_ *I*_1_(*t*) + *ρ*_1_ *I*_2_(*t*) with time after subtraction of the final term. When the energy broadening is in a Lorentzian form with an FWHM of Γ, the relaxation time τ of the exponential decay in *S*′(***q***, *t*) is written as *τ* = 2*ħ*/*Γ* ^1^. Note that the following time distributions must also be treated as incoherent broadening of the time spectrum: (i) the detector’s time resolution (in this case ~1 ns) and (ii) the SR arrival time distribution due to the spatial distribution of the electrons in a bunch (approximately 50 ps (FWHM) in the BL09XU beamline of SPring-8). As an experimental time spectrum, we obtained ${\bar{I}}_{\exp }({\boldsymbol{q}},t)=\bar{I}({\boldsymbol{q}},t)\otimes D(t)$, where *D*(*t*) is an incoherent distribution function obtained considering the above distributions.

The time spectrum is generally fitted based on the assumption of a relaxation function *F*(***q***, *t*), which represents the relaxation of *S*′(***q***, *t*) in the measurement time region around the time scale of *τ*_0_. Figure S2 in supplementary note §II shows an example of *F*(***q***, *t*). Here, we define the fitting parameter ${f}_{{\Gamma }_{0}}({\boldsymbol{q}})$ as ${f}_{{\Gamma }_{0}}({\boldsymbol{q}})\equiv \mathop{\mathrm{lim}}\limits_{t\to 0}F({\boldsymbol{q}},t)$. ${f}_{{\Gamma }_{0}}({\boldsymbol{q}})$ is the plateau value of *S*′(***q***, *t*) that is determined by fitting of the time spectrum in the measurement time window, as shown in Fig. S2. We recall that *f*_*ΔE*_(***q***) can also be determined via fitting using equation (2). When relaxation occurs in *S*(***q***, *t*) in the time range between *ΔT* and the initial time of the measurement time window, it therefore follows that ${f}_{\Delta E}({\boldsymbol{q}}) > {f}_{{\Gamma }_{0}}({\boldsymbol{q}})$. When there is a plateau within the time scale of *ΔT*, it can then be assumed that *f*_*ΔE*_(***q***) ~ *S*′(***q***, *ΔT*). Both *f*_*ΔE*_(***q***) and ${f}_{{\Gamma }_{0}}({\boldsymbol{q}})$ provide unique information about the microscopic dynamics, although particular attention should be paid to these definitions. Note here that the *f*_*ΔE*_(***q***) factor is hardly affected by the Compton scattering process under feasible experimental conditions, including the available sample, the gamma ray energies, and the scattering angles.

Here, we show that ${f}_{{\Gamma }_{0}}({\boldsymbol{q}})$ cannot be determined by conventional TDI methods using two identical emitters in the finite *ΔE* case, e.g., energies of several meV. When two identical emitters are used and one of these emitters is moved at a constant velocity with a resonant angular frequency shift of *Ω* ($\gg$ *Γ*_0_/*ħ*), we then obtain ${\bar{I}}_{\Delta E}^{{\rm{id}}}({\boldsymbol{q}},t)\propto {|G(t)|}^{2}\,\{1+{f}_{\Delta E}({\boldsymbol{q}})+2S^{\prime} ({\boldsymbol{q}},t)\,\cos \,(\Omega t)\}$ at *t* $\gg$ *ΔT* from equations (S5) and (S8). Additionally, when *ΔE* is sufficiently large and *f*_*ΔE*_(***q***) = 1 can be assumed, we then obtain

3 $${\bar{I}}_{\infty }^{{\rm{id}}}({\boldsymbol{q}},t)\propto {|G(t)|}^{2}\,\{1+S^{\prime} ({\boldsymbol{q}},t)\,\cos \,(\Omega t)\},$$

which is equivalent to the previously obtained equation^5–7^. The time spectrum that was obtained via conventional TDI was then fitted using equation (3) by assuming the function *F*(***q***, *t*), which represents the relaxation of *S*′(***q***, *t*) in the measurement time window.

However, the experiments to date have been performed under conditions where *ΔE* ranges up to several meV, which does not cover dynamic structure factor $\hat{S}({\boldsymbol{q}},\omega )$ sufficiently, as we show below, and is contrary to the assumption of the sufficiently large *ΔE* that was used to obtain the previous $\bar{I}({\boldsymbol{q}},t)$ ^5–7,9–11,17–24^. (See supplementary note §II for further details). In the finite *ΔE* case, rather than use ${\bar{I}}_{\infty }^{{\rm{id}}}({\boldsymbol{q}},t)$, ${\bar{I}}_{\Delta E}^{{\rm{id}}}({\boldsymbol{q}},t)$ should be used. We then rewrite the equation for ${\bar{I}}_{\Delta E}^{{\rm{id}}}({\boldsymbol{q}},t)$ as

4 $${\bar{I}}_{\Delta E}^{{\rm{id}}}({\boldsymbol{q}},t)\propto {|G(t)|}^{2}\,\{1+\frac{2S^{\prime} ({\boldsymbol{q}},t)}{1+{f}_{\Delta E}({\boldsymbol{q}})}\,\cos \,(\Omega t)\}.$$

When compared with equation (3), the cosine term has the additional factor 2/{1 + *f*_*ΔE*_(***q***)} in equation (4). When ${\bar{I}}_{\Delta E}^{{\rm{id}}}$ is used for the fitting, the function *F*′(***q***, *t*) is assumed for 2*S*′(***q***, *t*)/{1 + *f*_*ΔE*_(***q***)} as a coefficient of the cosine term. Using *F*(***q***, *t*), which represents the form of *S*′(***q***, *t*) in the measurement time window, *F*′(***q***, *t*) can be expressed as *F*′(***q***, *t*) = 2*F*(***q***, *t*)/{1 + *f*_*ΔE*_(***q***)}. It then follows that $\mathop{\mathrm{lim}}\limits_{t\to 0}F^{\prime} ({\boldsymbol{q}},t)=2{f}_{{\Gamma }_{0}}({\boldsymbol{q}})/\{1+{f}_{\Delta E}({\boldsymbol{q}})\}$. It is only in the case where *f*_*ΔE*_(***q***) = 1 that $\mathop{\mathrm{lim}}\limits_{t\to 0}F^{\prime} ({\boldsymbol{q}},t)$ has a simple physical meaning, as noted by Baron *et al*.^5^ Because both ${f}_{{\Gamma }_{0}}({\boldsymbol{q}})$ and *f*_*ΔE*_(***q***) are related to $\mathop{\mathrm{lim}}\limits_{t\to 0}F^{\prime} ({\boldsymbol{q}},t)$, then neither *f*_*ΔE*_(***q***) nor ${f}_{{\Gamma }_{0}}({\boldsymbol{q}})$ can be determined by conventional TDI methods (using an identical pair of emitters and incident SR with a finite energy width) in principle and the assumption *f*_*ΔE*_(***q***) = 1 thus cannot be validated from the spectrum itself. Conventional TDI therefore suffers from the uncertainty over the physical meaning of $\mathop{\mathrm{lim}}\limits_{t\to 0}F^{\prime} ({\boldsymbol{q}},t)$. In contrast, in the proposed multi-line method case, both *f*_*ΔE*_(***q***) and ${f}_{{\Gamma }_{0}}({\boldsymbol{q}})$ can be determined based on the difference between |*G*_1_(*t*)|^2^ and |*G*_2_(*t*)|^2^ because these values affect the spectrum in different ways, as indicated by equation (2).

**Figure 3:**
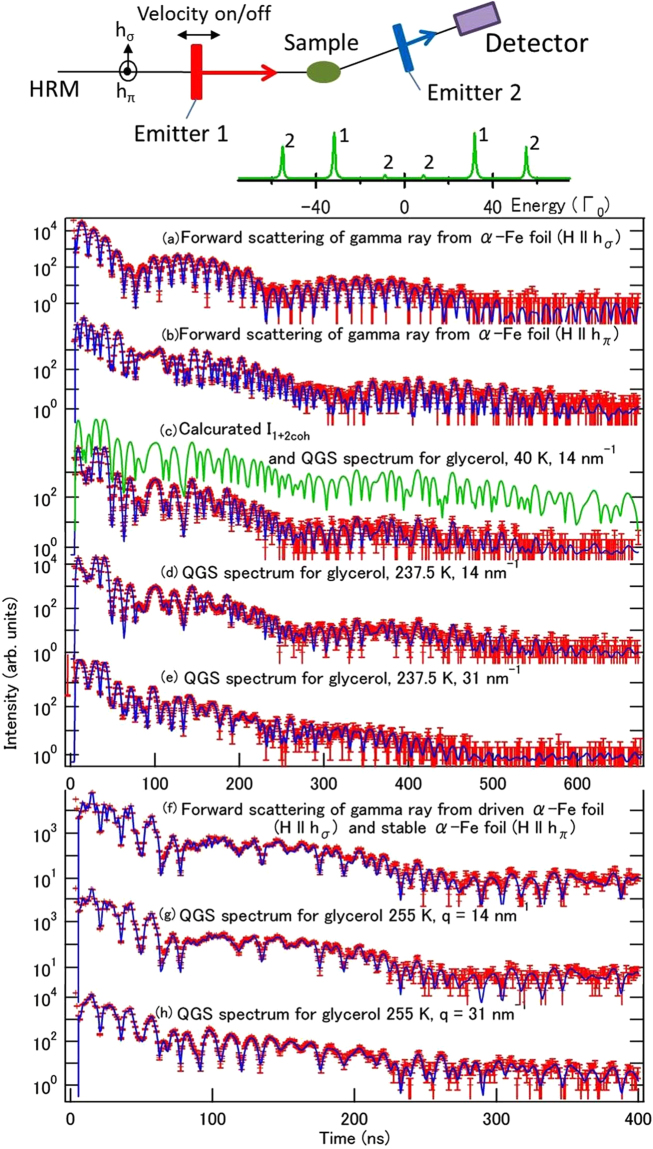
Schematic view of QGS setup using multi-line TDI, the time spectra obtained and their fitting curves. (Top) Schematic view of the experimental QGS setup using multi**-**line TDI is shown along with the energy spectrum of the gamma rays. Time spectra of the gamma rays: (**a**,**b**) obtained from calibration measurements in the forward direction from emitter 1 (α-Fe foil with ***H***‖***h***_**σ**_) and emitter 2 (α-Fe foil with ***H***‖***h***_**π**_); (**c**) obtained from the QGS experiment for glycerol at 237.5 K at 14 nm^−1^ (calculated time spectrum based on assumption of the results of the calibration measurements with both emitter 1 and emitter 2, which are also shown in the upper part as a green line); (**d**,**e**) obtained from the QGS experiments for glycerol at 237.5 K at 14 nm^−1^ and 31 nm^−1^, respectively, in the stable emitter condition; (**f**) obtained from the forward-scattering experiments with two α-Fe emitters in the driving condition; and (**g**,**h**) obtained from the QGS experiments for glycerol at 255 K at 14 nm^−1^ and 31 nm^−1^, respectively, in the driving emitter condition. In addition to these time spectra, fitting curves are also shown. Vertical bars denote the standard deviations of the measured points.

We consider the experimental setup and conditions shown in Fig. 3, where 14.4 keV gamma rays from stable α-iron foils are used; see the Methods section for full details. In this case, *δE*_12_ ~ 20 *Γ*_0_. Given our incident SR energy width of *ΔE* ~ 3.5 meV, we can then confirm the condition that ${\rm{\Delta }}E\gg \delta {E}_{12}\gg {\Gamma }_{0}$. However, when *Γ* $\mathop{ > }\limits_{ \tilde {}}$20 *Γ*_0_ (where the corresponding time scale is ~1  ns), the RC effect is not negligible. We can suppress the RC effect using the Doppler effect such that an energy shift of *E*_*γ*_ *v*/*c* is produced by driving the emitter at constant velocity *v*, where *c* is the speed of light and *E*_*γ*_ is the gamma ray energy. Note that the measurement efficiency for the dynamics is not greatly reduced by driving of the emitter if the time scale of the observed dynamics is equivalent to or shorter than that of the pseudo-relaxation. Here, as shown below, the pseudo-relaxation time is of the order of 100 ns. Therefore, when the relatively fast relaxation time $\tau \mathop{ < }\limits_{ \tilde {}}100\,{\rm{ns}}$ is considered as the target, driving does not seriously reduce the measurement efficiency and the multi-line system still shows much higher efficiency than the conventional system because of the second merit of the multi-line system (i.e. suppression of the speed-up effect). We refer to this setup as the driving emitter condition to distinguish it from the stable emitter condition described above. See supplementary note §III for further details of the driving condition.

## Results and Discussion

**Figure 4:**
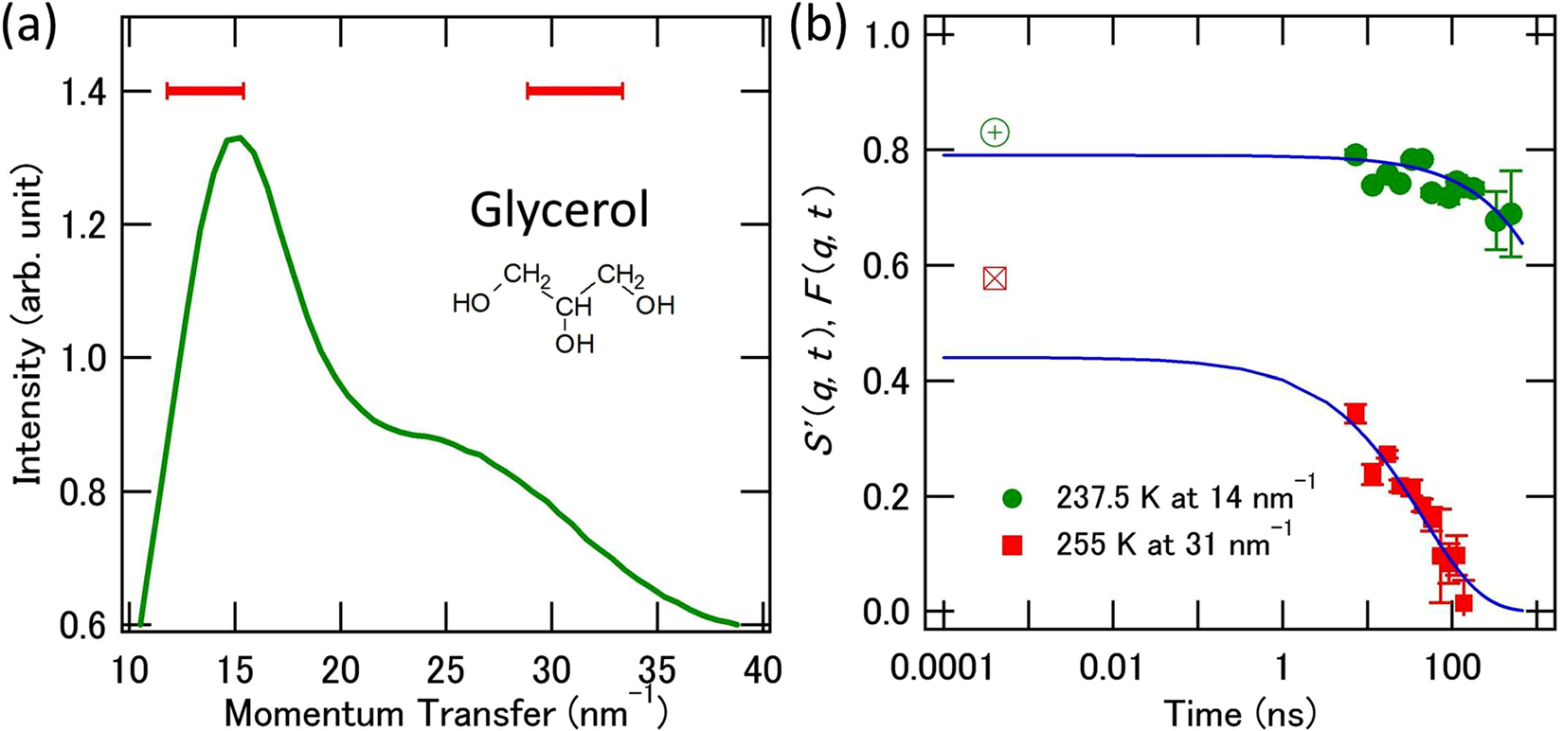
*q*-Dependence of the scattering intensity, and *f*_*ΔE*_ values and relaxation functions extracted from the QGS time spectra obtained for glycerol. (**a**) *q*-Dependence of the scattering intensity of glycerol at 250 K. Red bars indicate the *q* regions that are covered by the detectors. The molecular formula for glycerol is also shown. (**b**) *S*′(*q*, *t*) extracted from the time spectra and *F*(*q*, *t*) curves determined using the fitting results that were obtained for glycerol at 237.5 K at 14 nm^−1^ and at 255 K at 31 nm^−1^ are shown. The *f*_*ΔE*_ values obtained are plotted as circles with plus signs and squares with x-marks for the spectra at 237.5 K and 255 K, respectively.

The delayed time spectra that were obtained using the forward-scattering setups for α-Fe emitters 1 and 2 are shown in Fig. 3(a,b), respectively. In Fig. 3 and subsequent figures, vertical error bars denote the standard deviations of the values that were obtained. Because glycerol shows an isotropic *S*(***q***, *t*) form in the direction of ***q***, the dependence on *q =*|***q***| must then be considered. The *q*-dependence of the scattering intensity of glycerol at 250 K and the *q* region that was selected for the quasi-elastic scattering experiments are shown in Fig. 4(a). The *q*-dependence of the scattering intensity was hardly affected by the temperature in the temperature region of interest. Avalanche photodiode detectors (APDs) were placed at angles corresponding to the *q*-values that were related to the intermolecular and intramolecular scales at ~14 nm^−1^ and ~31 nm^−1^, respectively. The red bars indicate the *q* regions that were covered by the detectors. The measurement times were 2.5 h at 40 K, 5 h at 237.5 K and 2.5 h at 255 K. Figure 3(c) shows the QGS time spectrum that was obtained for glycerol at 40 K at 14 nm^−1^, where the molecular motions were too slow to be detected. We also show the QGS time spectra that were obtained at 237.5 K at 14 nm^−1^ and 31 nm^−1^ in Fig. 3(d,e), respectively. The forward-scattering time spectrum that was obtained using two α-Fe emitters under the driving condition is shown in Fig. 3(f). The QGS time spectra that were obtained for glycerol at 255 K at 14 nm^−1^ and 31 nm^−1^ under the driving condition are shown in Fig. 3(g,h), respectively.

As shown in Fig. 3(a,b), the forward-scattering time spectra can be fitted well using the MOTIF program^14^ with a magnetic hyperfine field of ~33 T, which is known to be the value of the α-Fe foil with only a slight modification because of the external magnetic field. The energy spectra of the gamma rays emitted from the α-Fe foils (emitters 1 and 2) are shown schematically in Fig. 3, where the numbers on the peaks indicate the emitters that are responsible for the corresponding peaks. By fitting these spectra, the emitter parameters that are required for the analysis of the QGS spectra can be obtained; *I*_1_(*t*), *I*_2_(*t*), and ${I}_{1+{2}_{{\rm{coh}}}}(t)$ are then calculated using these parameters. The calculated ${I}_{1+{2}_{{\rm{coh}}}}(t)$ is shown as the upper spectrum in Fig. 3(c). The spectrum that was obtained for glycerol at 40 K at 14 nm^−1^ is similar to the calculated ${I}_{1+{2}_{{\rm{coh}}}}(t)$ spectrum because the relaxation processes are suppressed well at low temperatures and ${I}_{1+{2}_{{\rm{coh}}}}(t)$ is the dominant component of the QGS time spectrum. When the emitter is driven, the pseudo-relaxation function *S* ^ps^(*t*) of ${I}_{1+{2}_{{\rm{coh}}}}^{{\prime}}(t)$ must be multiplied by *S*′(*q*, *t*) in equation (2). When fitting the spectrum, we assumed that *S* ^ps^(*t*) had the form exp{−(*t*/*τ* ^int^)^*m*^} because the Kohlrausch-Williams-Watts (KWW) function ${f}_{{\Gamma }_{0}}\,\exp \,\{-{(t/\tau )}^{{\beta }_{{\rm{KWW}}}}\}$ (where *β*_KWW_ is the stretching parameter) is appropriate as the relaxation function *F*(*q*, *t*); the pseudo-relaxation time *τ* ^int^ and *m* were determined to be 132 ns and 2.36, respectively.

Least squares fittings were performed for the QGS time spectra that were obtained under the stable emitter condition following equation (2) and using the calculated values of *I*_1_(*t*), *I*_2_(*t*), and ${I}_{1+{2}_{{\rm{coh}}}}(t)$. The mean relaxation time 〈τ〉 was then calculated from the obtained *τ* using 〈﻿*τ*﻿〉 = *τ*Γ(1/*β*_KWW_)/*β*_KWW_, where Γ is the gamma function. In the driving condition, the calculated *I*_1_(*t*), *I*_2_(*t*) and ${I}_{1+{2}_{{\rm{coh}}}}^{{\prime}}(t)$ were used to fit the QGS spectrum while taking *S* ^ps^(*t*) into account for the pseudo-broadening. Pseudo-relaxation of the sub-μs order (originating e.g. from external vibration) was determined via analysis of the QGS time spectrum at 40 K and was used to analyse the stable emitter condition. The fitting curves obtained are shown in Fig. 3(d,e). The 〈τ﻿﻿﻿﻿〉 and *β*_KWW_ results that were obtained at 237.5 K and 255 K for glycerol are shown in Table 1. Because the standard deviation of 〈τ﻿〉 that was obtained by fitting using a free *β*_KWW_ parameter only shows a large value in the 237.5 K case at 14 nm^−1^, the 〈τ〉 value that was obtained using the fixed *β*_KWW_ = 0.7 that was determined in a previous study^25^ is shown.

**Table 1:** 〈*τ*〉 and *β*_KWW_ values obtained for glycerol.

Temperature, *q*	〈*τ*〉 (ns)	*β*_KWW_
237.5 K, 14 nm^−1^	7704 ± 1354	Fix (0.7)
237.5 K, 31 nm^−1^	1213 ± 410	0.60 ± 0.16
255 K, 14 nm^−1^	282 ± 65	0.77 ± 0.20
255 K, 31 nm^−1^	66 ± 27	0.62 ± 0.26

In Fig. 4(b), we show that *S*′(*q*, *t*) was extracted from the QGS time spectrum by solving equation (2). Additionally, the *f*_*ΔE*_ value and the *F*(*q*, *t*) curves that were obtained by fitting of the QGS spectrum for glycerol at 237.5 K at 14 nm^−1^ and at 255 K at 31 nm^−1^ are shown with the time scale *ΔT* to allow visual comparison of *f*_*ΔE*_ and ${f}_{{\Gamma }_{0}}$. The results of fitting *S*′(*q*, *t*) by *F*(*q*, *t*) can be confirmed visually from the figure. The *τ* values that were obtained at 14 nm^−1^ at 237.5 K and 255 K are consistent with previous dielectric spectroscopy and quasi-elastic neutron scattering results^25,26^. Our results showed minor *q* and temperature dependences for *β*_KWW_ ~ 0.7 within the studied *q* and deeply supercooled temperature regions, where the *β*_KWW_ value has not previously been determined via quasi-elastic scattering experiments. When using conventional single-line TDI, the *β*_KWW_ values could not be determined using one-day measurements, and this indicates that the measurement efficiency is significantly improved when using multi-line TDI in stable conditions and even in driving emitter conditions.

Finally, we must mention the *f*_*ΔE*_ and ${f}_{{\Gamma }_{0}}$ that were obtained. We found that the relationship ${f}_{\Delta E}\sim {f}_{{\Gamma }_{0}}$ holds at 40 K with precision of a few percent at both *q* values (14 nm^−1^ and 31 nm^−1^). This result confirms that the microscopic relaxation process hardly occurs within a time scale ranging from sub-picoseconds to several nanoseconds at 40 K^25^. It was also confirmed that the relation *f*_*ΔE*_ > ${f}_{{\Gamma }_{0}}$ holds at both 237.5 K and 255 K for both *q* values (14 nm^−1^ and 31 nm^−1^). As examples of the obtained *f*_*ΔE*_ and ${f}_{{\Gamma }_{0}}$ values, we show the results that were obtained at 255 K: at 14 nm^−1^, *f*_*ΔE*_ = 0.85 ± 0.01, ${f}_{{\Gamma }_{0}}=0.72\pm 0.03$, while at 31 nm^−1^, *f*_*ΔE*_ = 0.56 ± 0.01, ${f}_{{\Gamma }_{0}}=0.45\pm 0.04$. In a relatively fast time region, *S*(*q*, *t*) was measured for glycerol in a quasi-elastic neutron scattering experiment and was also obtained via simulations^27,28^. The results show that faster processes such as the fast *β* process contribute to the decay of *S*(*q*, *t*) on a sub-ps time scale^27,28^. Therefore, the relation *f*_*ΔE*_ > ${f}_{{\Gamma }_{0}}$ that was observed in our experiments is explained by the existence of the fast processes. For comparison with the normalized RSMR results, the ${f}_{{\Gamma }_{0}}$ values that were obtained at 14 nm^−1^ at 255 K were normalized with respect to the ${f}_{{\Gamma }_{0}}$ value that was obtained at the low temperature of 40 K^29^. The normalized ${f}_{{\Gamma }_{0}}$ value of 0.96 ± 0.01 at 14 nm^−1^ at 255 K is consistent with the normalized RSMR results^29^ and thus validates our measurement results and analyses.

## Conclusions

In this report, we generalize the expression for the QGS spectrum using TDI in the QB regime with an identical pair of gamma-ray emitters to the case with a nonidentical gamma-ray emitter pair. We modified the expression based on consideration of the effects of the finite energy width (typically meV) of the incident SR. The resulting expression illustrates the unique characteristics of the multi-line QGS spectra. Glycerol was studied using 14.4 keV gamma rays from ^57^Fe nuclei. A normalized intermediate scattering function was extracted from the resulting spectra and a relaxation time of the order of 1 μs was obtained, even at a relatively high *q* ~ 31 nm^−1^. The stretching parameter of the KWW function could be determined, even in a deeply supercooled state in the high *q* region. The multi-line QGS method produces the microscopic relaxation picture more rapidly and accurately than conventional single-line QGS. Compensative usage of QGS using TDI and SMS combined with other methods such as quasi-elastic neutron scattering is expected to produce detailed dynamic pictures of condensed matter.

## Methods

QGS experiments using multi-line TDI with 14.4 keV Mössbauer gamma-rays from ^57^Fe nuclei in the first nuclear state excited by σ-polarized SR were performed on a nuclear resonant scattering beamline (BL09XU) at SPring-8 in Japan. The storage ring was operated in a several-bunch timing mode (1/7-filling + 5-bunch mode with a bunch interval of 684.3 ns) at a current of 100 mA. The incident SR was monochromatized to *ΔE* ~ 3.5 meV using a high-resolution monochromator consisting of asymmetric Si (5 1 1) and Si (9 7 5) channel-cut crystals near the excitation energy. ^57^Fe-enriched (>96%) α-Fe foils with nominal thicknesses of 3 μm and 4 μm were used for emitters 1 and 2, respectively. We applied an external magnetic field ***H*** of ~0.6 T to the upstream and downstream foils in different directions (as shown in Fig. 3) to select the allowed transitions between the nuclear energy levels that were split by the hyperfine interaction. The polarization vectors of the magnetic field of the incident SR are written as ***h***_**σ**_ and ***h***_**π**_ when they are directed parallel and perpendicular to the scattering plane, respectively. Under the condition where ***H***‖***h***_**σ**_, two transitions (Δ*m* = 0; Δ*m* denotes the magnetic quantum number difference between ground and the excited ^57^Fe nucleus) are allowed and gamma rays with two different energies are emitted. Conversely, the four remaining transitions (Δ*m* = ±1) are allowed under the condition ***H***‖***h***_**π**_. The sample temperature was controlled using a He-flow cryostat. The melting and glass transition temperatures of glycerol are 291 K and 188 K, respectively. A double-stacked eight-element Si APD detector was used. Each detector element had a surface area of 3 × 5 mm^2^ with a 4 × 2 array alignment (giving dimensions of 12 × 10 mm^2^ in total). The alignment of these elements in the detector is shown in Fig. S3 in supplementary note §III. The depletion layer was approximately 120 μm thick. Time spectra were measured using a fast multi-channel scaler (MCS6, FAST ComTec GmbH, München, Germany). The measurements of glycerol were performed at temperatures of 40 K and 237.5 K under the stable emitter condition. The measurements of glycerol at 255 K were performed under the driven upstream emitter condition using a Mössbauer velocity transducer (MVT-1000, WissEl GmbH, Starnberg, Germany) at a constant velocity of ~20.4 mm/s (giving an energy shift of 212 *Γ*_0_), which is sufficient for observation of the relaxation times obtained in this study.

### Data availability

The datasets generated during and/or analysed during the current study are available from the corresponding author on reasonable request.

## Electronic supplementary material

supplementary note
