# Supplementary material for: Synchrotron radiation-based quasi-elastic scattering using time-domain interferometry with multi-line gamma rays
Source: Sci Rep. 2017 Oct 2;7:12558. doi: 10.1038/s41598-017-12216-7 (PMC5624928; doi:10.1038/s41598-017-12216-7)
Supplement: Supplementary file 1 — supplementary note [file 41598_2017_12216_MOESM1_ESM.pdf]

Synchrotron radiation-based quasi-elastic scattering using time-domain interferometry with multi-line gamma rays

Makina Saito, Ryo Masuda, Yoshitaka Yoda, and Makoto Seto

## Supplementary Note

### I. Examples of dynamic studies performed through RSMR, QGS, and Mössbauer experiments

To date, RSMR, QGS and Mössbauer spectroscopy methods have been used to study the dynamics of materials such as supercooled liquids, solids, liquid crystals and biomolecules. It has been shown that in-depth study of the microscopic dynamics of supercooled liquids is critical to understanding of the nature of the glass transition. Supercooled liquids gradually assume the microscopic characteristics of glass over a time scale of approximately 100 ns[S1,S2], which is covered by QGS. The use of QGS has been reported in dynamic studies of *o*-terphenyl,[S3-S5] polybutadiene,[S5] glycerol[S4,S6] and ionic liquid 1-butyl-3-methylimidazolium iodide[S7,S8] to date. In solids, RSMR has been used to study the soft modes of $BaTiO_3$[S9] and $SrTiO_3$[S10,S11] and has indicated that structural changes occur on time scales of more than 100 ns at the temperatures studied. In addition, the plastic crystal-crystal phase transition has been studied using RSMR.[S12] Diffusion in ordered alloys has also been studied using QGS.[S13,S14] The time scale of the molecular translational dynamics of liquid crystal systems is also covered by QGS, as indicated by probe experiments using Mössbauer spectroscopy.[S15-S17] A typical thermotropic liquid crystal and a partially fluorinated amphiphilic liquid crystal were measured using QGS and their anisotropic relaxation times were also studied.[S18] Additionally, the dynamics of protein-water systems have been studied via RSMR,[S19] and the dynamics of both colloidal systems and proteins have been studied in probe experiments using Mössbauer spectroscopy.[S20,S21]

### II. Derivation of expression for the QGS time spectrum using TDI

The time variation of $|G_i(t)|$ is much slower than that of $|E_0(t)|$ because of the relation $\tau_0 \gg \delta T_{12} \gg \Delta T$, and it therefore follows for the first term in equation (1) that $-g(\boldsymbol{q}, t_s + t)\int_{-\infty}^{\infty} dt' G_1(t-t')\, E_0(t') \cong -g(\boldsymbol{q}, t_s + t) G_1(t) \int_{-\infty}^{\infty} dt'\, E_0(t')$. Similarly, the second term can be written as $\int_{-\infty}^{\infty} dt' G_2(t-t') g(\boldsymbol{q}, t_s + t')\, E_0(t') \cong G_2(t) \int_{-\infty}^{\infty} dt' g(\boldsymbol{q}, t_s + t')\, E_0(t')$. Here, we define $g_c(\boldsymbol{q}, t_s + t)$ as $g_c(\boldsymbol{q}, t_s + t) \equiv \int_{-\infty}^{\infty} dt'\, g(\boldsymbol{q}, t_s + t + t') E_0(t') / \int_{-\infty}^{\infty} dt'\, E_0(t')$. When $E_0(t) = \delta(t)$, it then naturally follows that $g_c(\boldsymbol{q}, t_s + t) = g(\boldsymbol{q}, t_s + t)$. Using $g_c$, equation (1) can then be rewritten as

$$E_C^{delay}(\boldsymbol{q}, t, t_s) \cong -\sqrt{\rho_1 \rho_2}\, \hat{E}_0(0) \{ g(\boldsymbol{q}, t_s + t) G_1(t) + G_2(t) g_c(\boldsymbol{q}, t_s) \}, \quad \text{(S1)}$$

where we used the general relation of the Fourier transformation $\int_{-\infty}^{\infty} dt' E_0(t') = \hat{E}_0(0)$. Here, $g_c(\boldsymbol{q}, t_s)$ represents the integration of the product of $g(\boldsymbol{q}, t_s + t')$ and $E_0(t')$ with respect to $t'$. This integration is caused by the complex situation shown in paths II, where the gamma rays that are scattered by the sample at various times denoted by $t_s + t'$ ($|t'| \lesssim \varDelta T$) interfere with each other at the detector position at $t$, as shown in Fig. S1(b). Because of this integration, information about any dynamics that are much faster than $\varDelta T$ is lost in the second term, as shown below.

Fig. S1

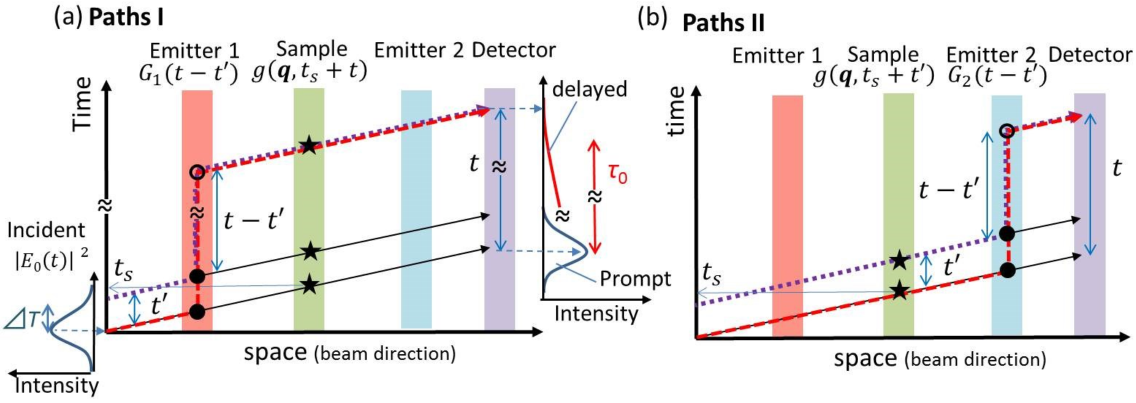


**Figure S1. Time-space diagrams of gamma-ray paths with different incident times.**
Examples of the paths of incident SR and gamma rays emitted by (a) emitter 1 and (b) emitter 2. Gamma rays that were detected at $t$ with incident times of $t' = 0$ (long dashed lines) and $t' \neq 0$ (short dashed lines) are shown. Bold lines indicate the paths of the prompt SR. The gamma ray detection time $t$ is around the time scale of $\tau_0$; in contrast, the incident time $t'$ is distributed over a much shorter time scale where $|t'| \lesssim \varDelta T$.

We can then obtain the delayed signal's intensity $I(\boldsymbol{q}, t, t_s) = \left|E_C^{delay}(\boldsymbol{q}, t, t_s)\right|^2$:

$$I(\boldsymbol{q}, t, t_s) \propto |G_1(t)|^2 |g(\boldsymbol{q}, t_s + t)|^2 + |G_2(t)|^2 |g_c(\boldsymbol{q}, t_s)|^2$$

$$+ G_1^*(t) G_2(t) g^*(\boldsymbol{q}, t_s + t) g_c(\boldsymbol{q}, t_s) + G_1(t) G_2^*(t) g(\boldsymbol{q}, t_s + t) g_c^*(\boldsymbol{q}, t_s). \quad \text{(S2)}$$

The observed $\bar{I}(\boldsymbol{q}, t)$ is obtained by averaging $I(\boldsymbol{q}, t, t_s)$ with respect to $t_s$ over the long measurement time $t_m$.[S22-S24] In a system without long-range ordering, $S(\boldsymbol{q}, t)$ can then be written as $S(\boldsymbol{q}, t) = \frac{1}{t_m} \int dt_s\, g^*(\boldsymbol{q}, t_s + t) g(\boldsymbol{q}, t_s) = \frac{1}{t_m} \int dt_s\, g(\boldsymbol{q}, t_s + t) g^*(\boldsymbol{q}, t_s)$,[S23-S25,S26] where $S(\boldsymbol{q}, t)$ is a real number that is derived from the correlation function characteristics.[S26] Similarly, we can define the correlation functions $S_{cc}(\boldsymbol{q}, t)$ and $S_c(\boldsymbol{q}, t)$ as $g_c^*(\boldsymbol{q}, t_s) g_c(\boldsymbol{q}, t_s + t)$ and $g^*(\boldsymbol{q}, t_s + t) g_c(\boldsymbol{q}, t_s)$ when averaged with respect to $t_s$, respectively. $S_{cc}(\boldsymbol{q}, t)$ can also be shown to be a real number. The observed time-averaged intensity $\bar{I}(\boldsymbol{q}, t)$ can be written as

$$\bar{I}(\boldsymbol{q}, t) \propto S(\boldsymbol{q}, 0) |G_1(t)|^2 + S_{cc}(\boldsymbol{q}, 0) |G_2(t)|^2 + S_c(\boldsymbol{q}, t) G_1^*(t) G_2(t) + S_c^*(\boldsymbol{q}, t) G_1(t) G_2^*(t). \quad \text{(S3)}$$

Here, $S_c(\boldsymbol{q},t)$ is written as

$$S_c(\boldsymbol{q},t)=\frac{1}{t_m}\int dt_s\, g^*(\boldsymbol{q},t_s+t)g_c(\boldsymbol{q},t_s)=\frac{1}{\hat{E}_0(0)}\int_{-\infty}^{\infty}dt'\,E_0(t')S(\boldsymbol{q},t-t'). \quad \text{(S4)}$$

The time spectrum is assumed to be measured over a much longer time scale than $\varDelta T$, and in the measurement time window, the variation of $S(\boldsymbol{q},t)$ with the time scale $\varDelta T$ is negligible in conventional condensed matter. In this case, it can be assumed that the effect of the convolution in equation (S4) on the form of $S(\boldsymbol{q},t)$ is negligible. It therefore follows that $S_c(\boldsymbol{q},t)\cong S(\boldsymbol{q},t)\int_{-\infty}^{\infty}dt'E_0(t')/\hat{E}_0(0)\cong S(\boldsymbol{q},t)$ on a time scale that is much longer than $\varDelta T$, and $S_c(\boldsymbol{q},t)\cong S_c^*(\boldsymbol{q},t)$. $\bar{I}(\boldsymbol{q},t)$ is then written as follows:

$$\bar{I}(\boldsymbol{q},t)\propto S(\boldsymbol{q},0)|G_1(t)|^2+S_{cc}(\boldsymbol{q},0)|G_2(t)|^2+S(\boldsymbol{q},t)[G_1^*(t)G_2(t)+G_2^*(t)G_1(t)]\ \ (\text{at } t\gg\varDelta T). \quad \text{(S5)}$$

As shown in Fig. S1(a), all of paths I cross the sample at the same time: $t_s+t$. Consequently, in the first term of equation (S5), $|G_1(t)|^2$ is linear with respect to $S(\boldsymbol{q},0)$. In contrast, the $S_{cc}(\boldsymbol{q},0)$ factor of the second term in equation (S5) is derived from the complex interference of the gamma rays as they pass through paths II. From the definition of $S_{cc}(\boldsymbol{q},t)$, it then follows that

$$S_{cc}(\boldsymbol{q},t)=\frac{1}{|\hat{E}_0(0)|^2}\frac{1}{t_m}\int dt_s\int_{-\infty}^{\infty}dt'\int_{-\infty}^{\infty}dt''\,{E_0}^*(t')E_0(t'')g^*(\boldsymbol{q},t_s+t')\,g(\boldsymbol{q},t_s+t+t'')$$
$$=\int_{-\infty}^{\infty}dt'''I_0(t''')S(\boldsymbol{q},t+t''') \quad \text{(S6)}$$

where $t'''\equiv t''-t'$, and we define $I_0(t''')\equiv\int_{-\infty}^{\infty}dt'\,{E_0}^*(t')E_0(t'''+t')/|\hat{E}_0(0)|^2$. The typical time scale for the decay of $I_0(t''')$ is denoted by $\varDelta T$. When $t\gg\varDelta T$, it follows that $S_{cc}(\boldsymbol{q},t)\sim S(\boldsymbol{q},t)$ when the time variation of $S(\boldsymbol{q},t)$ with the time scale $\varDelta T$ is neglected, as assumed in the discussion of $S_c(\boldsymbol{q},t)$ above; conversely, when $t\ll\varDelta T$, e.g. at $t=0$, it then follows that $S_{cc}(\boldsymbol{q},0)=\int_{-\infty}^{\infty}dt'''I_0(t''')S(\boldsymbol{q},t''')$.

For further analysis of the meaning of $S_{cc}(\boldsymbol{q},0)$, which appears in the second term of equation (S5), we must also consider the frequency-domain view. Using $\hat{I}_0(\omega)=|\hat{E}_0(\omega)|^2/|\hat{E}_0(0)|^2$, which is the frequency-domain representation of $I_0(t)$, the frequency-domain representation $\hat{S}_{cc}(\boldsymbol{q},\omega)$ can be written as:

$$\hat{S}_{cc}(\boldsymbol{q},\omega)=\int_{-\infty}^{\infty}dte^{i\omega t}\,S_{cc}(\boldsymbol{q},t)=\hat{I}_0(-\omega)\hat{S}(\boldsymbol{q},\omega), \quad \text{(S7)}$$

where $\hat{S}(\boldsymbol{q},\omega)\equiv\int_{-\infty}^{\infty}dt\,\mathrm{e}^{i\omega t}S(\boldsymbol{q},t)=|\hat{g}(\boldsymbol{q},\omega)|^2$ is the frequency-domain representation of $S(\boldsymbol{q},t)$ and is called the dynamic structure factor.[S26] $S_{cc}(\boldsymbol{q},0)$ is written as $S_{cc}(\boldsymbol{q},0)=\int_{-\infty}^{\infty}d\omega\,\hat{S}_{cc}(\boldsymbol{q},\omega)=\int_{-\infty}^{\infty}d\omega\,\hat{I}_0(-\omega)\hat{S}(\boldsymbol{q},\omega)$. Here, we define the value $f_{\varDelta E}(\boldsymbol{q})$ as

$$f_{\varDelta E}(\boldsymbol{q})\equiv S_{cc}(\boldsymbol{q},0)/S(\boldsymbol{q},0)=\int_{-\infty}^{\infty}d\omega\,\hat{I}_0(-\omega)\hat{S}(\boldsymbol{q},\omega)/\int_{-\infty}^{\infty}d\omega\,\hat{S}(\boldsymbol{q},\omega). \quad \text{(S8)}$$

In general, $f_{\varDelta E}(\boldsymbol{q})$ represents the part of $\hat{S}(\boldsymbol{q},\omega)$ that is covered by the energy spectrum of the incident SR $\hat{I}_0(-\omega)$. When the primary part of the central peak of $\hat{S}(\boldsymbol{q},\omega)$ is covered sufficiently by $\hat{I}_0(-\omega)$ and the side-band peaks that are related to e.g., the phonons are hardly covered by $\hat{I}_0(-\omega)$, $f_{\varDelta E}(\boldsymbol{q})$ can

then be treated as the Debye-Waller factor. $S_{cc}(\boldsymbol{q},0)$ and $f_{\Delta E}(\boldsymbol{q})$ are therefore affected by the energy width of the incident SR.

Fig. S2

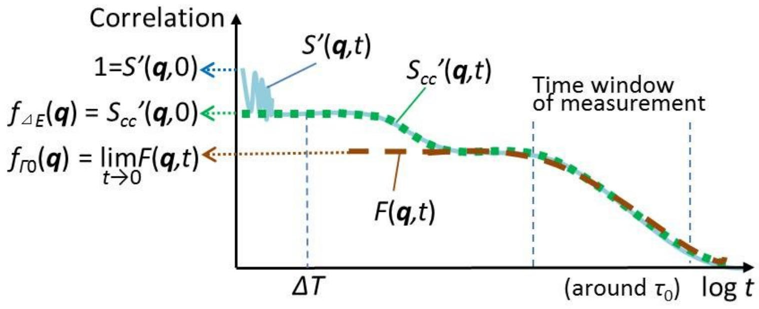


**Figure S2. Examples of relationships among $S'(\boldsymbol{q},t)$, $S'_{cc}(\boldsymbol{q},t)$, and $F(\boldsymbol{q},t)$.**

Examples of $S'(\boldsymbol{q},t)$ and $S'_{cc}(\boldsymbol{q},t)$ are shown, based on the assumption that the lattice vibrations occur on a timescale that is faster than $\Delta T$, along with two relaxation processes that occur (1) in the time between $\Delta T$ and the fast limit of the measurement time window and (2) in the time measurement window. At times much longer than $\Delta T$, it follows that $S'(\boldsymbol{q},t) \sim S'_{cc}(\boldsymbol{q},t)$. In addition, the function $F(\boldsymbol{q},t)$, which is used to represent $S'(\boldsymbol{q},t)$ in the fitting procedure, is also shown.

$S'(\boldsymbol{q},t)$ generally shows a multi-step relaxation form that is spread over broad time ranges, as will be discussed further in the glycerol case in the main text. Fig. S2 shows an example of $S'(\boldsymbol{q},t)$ with a vibration process and several relaxation processes along with its relationship to $S'_{cc}(\boldsymbol{q},t) \equiv S_{cc}(\boldsymbol{q},t)/S(\boldsymbol{q},0)$. The time spectrum is usually fitted via the assumption of the relaxation function $F(\boldsymbol{q},t)$, which represents the relaxation of $S'(\boldsymbol{q},t)$ in the measurement time region in the time scale around $\tau_0$. Fig. S2 also shows an example of $F(\boldsymbol{q},t)$.

**III. Details of the driving condition and the experimental alignment of the detector**

In the driving condition, the QGS time spectrum is also expressed using equation (2), but $I'_{1+2_{\mathrm{coh}}}(t)$ is used rather than $I_{1+2_{\mathrm{coh}}}(t)$, where $I'_{1+2_{\mathrm{coh}}}(t)$ expresses the time spectrum of the gamma rays that were obtained using the forward-scattering measurement setup in the driving condition. If $\Omega$ is suitably large and the relation ($\Delta E \gg$) $\delta E_{12} \gg \Gamma$ holds for all gamma ray line pairs, then the RC effect is negligible. Note that the shapes of $I_1(t)$ and $I_2(t)$ are not changed by the driving condition. In the $I'_{1+2_{\mathrm{coh}}}(t)$ time spectrum, a pseudo-relaxation effect similar to the relaxation results from the driving condition, typically on a time scale of 100 ns (where the corresponding energy broadening is $\sim\Gamma_0$), in the setup shown in the main text. In the case where $\Gamma > \Gamma_0$, in which relaxation occurs on a shorter time scale than the pseudo relaxation, $\sim\Gamma_0$, the measurement efficiency is not greatly reduced. Therefore, it is

proposed that the driving emitter condition is favourable when fast dynamics with $\Gamma > \Gamma_0$ are expected; otherwise, stable emitter conditions produce better measurement efficiency.

Fig. S3 shows a schematic of the experimental alignment of the detector with the double-stacked eight-element Si APD array. Using this alignment scheme, the time spectra can be measured at four $q$ points simultaneously using one detector.

Fig. S3

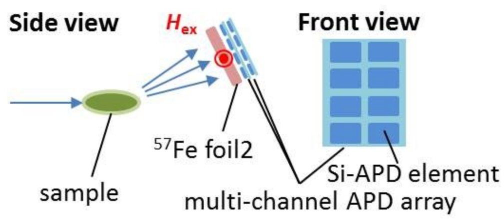


**Figure S3. Schematic figure of APD detector.**
Experimental alignment of the detector with the double-stacked eight-element Si APD array is shown. Each APD element had a surface area of 3×5 $mm^2$ with alignment in a 4×2 array (giving dimensions of 12×10 $mm^2$ in total) with 1 mm intervals between the elements, as indicated by the front view.

**References**

S1 Fujara, F., Geil, B., Sillescu, H., & Fleischer, G. Z. Translational and rotational diffusion in supercooled orthoterphenyl close to the glass transition. *Phys. B Cond. Matt.* **88** 195–204 (1992).

S2 Novikov, V. N., & Sokolov, A. P. Universality of the dynamic crossover in glass-forming liquids: a “magic” relaxation time. *Phys. Rev. E* **67** 031507 (2003).

S3 Saito, M. *et. al.* Slow processes in supercooled o-terphenyl: relaxation and decoupling. *Phys. Rev. Lett.* **109** 115705 (2012).

S4 Saito, M. *et. al.* Slow dynamics of supercooled liquid revealed by Rayleigh scattering of Mössbauer radiation method in time domain. *Hyperfine Interact.* **226** 629–636 (2014).

S5 Kanaya, T., Inoue, R., Saito, M., Seto, M., & Yoda, Y. Relaxation transition in glass-forming polybutadiene as revealed by nuclear resonance X-ray scattering. *J. Chem. Phys.* **140** 144906 (2014).

S6 Saito, M. *et. al.* Slow dynamics in glycerol: collective de Gennes narrowing and independent angstrom motion. *Hyperfine Interact.* **237** 22 (2016).

S7 Saito, M. *et. al.* Development of time-domain interferometry for the study of glass formers. *J. Phys.: Conf. Ser.* **217** 012147 (2010).

S8 Saito, M. *et. al.* Development of 151-Eu time-domain interferometry and its application for the study of slow dynamics in ionic liquids. *Appl. Phys. Express* **2** 026502 (2009).

S9 O'Connor, D. A., & Spicer, E. R. The scattering of gamma rays by optical phonons in barium titanate. *Phys. Lett.* **29A** 136–137 (1969).

S10 Darlington, C. N. W., Fitzgerald, W. J., & O'Connor, D. A. On the energy width of the central mode in the critical scattering of X-rays by $SrTiO_3$. *Phys. Lett.* **54A** 35–36 (1975).

S11 Darlington, C. N. W., & O'Connor, D. A. The central mode in the critical scattering of X-rays by $SrTiO_3$. *J. Phys. C: Solid State Phys.* **9** 3561–3571 (1976).

S12 Champeney, D. C., & Sedgwick, D. F. The molecular dynamics of solid cyclohexane by Mössbauer scattering. *J. Phys. C: Solid St. Phys.* **4** 2220–2222 (1971).

S13 Sepiol, B. *et. al.* Quasielastic scattering of synchrotron radiation from non-resonant atoms. *Hyperfine Interact.* **126** 329–333 (2000).

S14 Kaisermayr, M. *et. al.* Time-domain interferometry using synchrotron radiation applied to diffusion in ordered alloys. *Eur. Phys. J. B* **20** 335–341 (2001).

S15 Uhrich, D. L., Wilson, J. M., & Resch, W. A. Mössbauer investigation of the smectic liquid crystalline state. *Phys. Rev. Lett.* **24** 355–359 (1970).

S16 LaPrice, W. J., & Uhrich, D. L. A Mössbauer temperature study of a cold nematic liquid crystal: nematic glass–supercooled nematic. *J. Chem. Phys.* **71** 1498–1505 (1979).

S17 LaPrice, W. J., & Uhrich, D. L. A Mössbauer temperature study of two cold smectic (B and H) liquid crystals. *J. Chem. Phys.* **72** 678–686 (1980).

S18 Saito, M. *et. al.* Small and large angle quasi-elastic scattering experiments by using nuclear resonant scattering on typical and amphiphilic liquid crystals. *J. Phys. Soc. Jpn.* **81** 023001 (2012).

S19 Krupyanskii, Yu. F., Goldanskii, V. I., Nienhaus, G. U., & Parak, F. Dynamics of protein-water systems revealed by Rayleigh scattering of Mössbauer radiation (RSMR). *Hyperfine Interact.* **53** 59–73 (1990).

S20 Hendriksen, P. V., Mørup, S., & Linderoth, S. Brownian oscillations in colloidal systems studied by Mossbauer spectroscopy. *J. Phys.: Cond. Matt.* **3** 3109–3124 (1991).

S21 Young, R. D., Frauenfelder, H., & Fenimore, P. W. Mössbauer effect in proteins. *Phys. Rev. Lett.* **107** 158102 (2011).

S22 Hastings, J. B., Siddons, D. P., van Bürck, U., Hollatz, R., & Bergmann, U. Mössbauer spectroscopy using synchrotron radiation. *Phys. Rev. Lett.* **66** 770–773 (1991).

S23 Baron, A. Q. R. *et. al.* Quasielastic scattering of synchrotron radiation by time domain interferometry. *Phys. Rev. Lett.* **79** 2823–2826 (1997).

S24 Smirnov, G. V., Kohn, V. G., & Petry, W. Dynamics of electron density in a medium revealed by Mössbauer time-domain interferometry. *Phys. Rev. B* **63** 144303 (2001).

S25 Smirnov, G. V. *et. al.* Nuclear $\gamma$ resonance time-domain interferometry: quantum beat and radiative coupling regimes compared in revealing quasielastic scattering. *Phys. Rev. B* **73** 184126 (2006).

S26 Balucani, U., & Zoppi, M. *Dynamics of the Liquid State*. (Clarendon press, 1994).
